# Functional and non-functional pancreatic neuroendocrine tumours: ENETS or AJCC TNM staging system?

**DOI:** 10.18632/oncotarget.20007

**Published:** 2017-08-07

**Authors:** Min Yang, Neng-Wen Ke, Yi Zhang, Chun-Lu Tan, Bo-Le Tian, Xu-Bao Liu, Wei Huang, Quentin Nunes, Robert Sutton

**Affiliations:** ^1^ Department of Pediatric Surgery, West China Hospital, Sichuan University, Chengdu, China; ^2^ Department of Pancreatic Surgery, West China Hospital, Sichuan University, Chengdu, China; ^3^ Department of Integrated Traditional Chinese and Western Medicine, Sichuan Provincial Pancreatitis Centre, West China Hospital, Sichuan University, Chengdu, China; ^4^ National Institute for Health Research (NIHR) Liverpool Pancreas Biomedical Research Unit, Royal Liverpool University Hospital, University of Liverpool, Liverpool, UK

**Keywords:** pancreatic neuroendocrine tumours, functional status, TNM, ENETS, AJCC

## Abstract

**Background:**

There are currently 2 Tumour-Node-Metastasis (TNM) staging systems for pancreatic neuroendocrine tumours (p-NETs) - European Neuroendocrine Tumour Society (ENETS) and American Joint Committee on Cancer (AJCC). P-NETs being heterogeneous, we investigated the prognostic value of the 2 systems in p-NETs, as a whole, and more interestingly in functional and non-functional sub-groups separately, with a view to ascertaining any potential clinical benefits of using one system over the other.

**Methods:**

Data from patients with surgically resected p-NETs were retrospectively reviewed. Kaplan-Meier method and Cox Regression proportional hazards model were used to analyse overall survival (OS) and prognostic predictors respectively.

**Results:**

In the whole group of 165 patients, both TNM systems successfully discriminated OS differences when comparing stages I and II with stages III and IV (*P*<0.05); ENETS stage III patients had a significantly better OS than those in stage IV (*P*=0.003). Patients with functional p-NETs in ENETS stage II showed a statistically better OS than those in stages III and IV (*P*<0.05). For non-functional tumours, the AJCC staging system could effectively discriminate between the OS differences of patients in stage I with stages III and IV, or stage II with III and IV (*P*<0.05). Along with surgical intent and World Health Organisation (WHO) 2010 grade, both ENETS and AJCC staging systems were effective predictors of OS for different function-status p-NETs.

**Conclusions:**

The ENETS system might have potential advantages when applied to all p-NETs and to the functional sub-group, while the AJCC system might be clinically more practical for non-functional p-NETs.

## INTRODUCTION

Pancreatic neuroendocrine tumours (p-NETs) are rare, accounting for <3% of all pancreatic tumours with an annual incidence of approximately <1 per 100,000 population [1-4]. They have shown an increasing prevalence over the past two decades largely due to widespread awareness and advances in diagnostic techniques [2-6]. P-NETs may produce a variety of hormones, such as insulin, gastrin, glucagon, vasoactive intestinal peptide (VIP), serotonin, somatostatin, adrenocorticotropic hormone (ACTH), etc. and are regarded as functional when hormone overproduction is associated with clinical features of the same [7]. In contrast, p-NETs without corresponding symptoms of hormone overproduction are considered to be non-functional, which may present with abdominal pain, jaundice, weight loss or other non-specific symptoms or may be detected incidentally [6, 8, 9]. Now, there are no universally accepted definitions of functional and non-functional p-NETs [3, 10, 11].

Due to their rarity and heterogeneous nature, p-NETs have been poorly defined, and the ability to classify patients with p-NETs into prognostic groups has always been challenging [6]. At present, there are two main Tumour-Node-Metastasis (TNM) systems to stage p-NETs. The European Neuroendocrine Tumour Society (ENETS) staging system [12] was proposed by Rindi et al. in 2006 for gastrointestinal and pancreatic neuroendocrine tumours. The American Joint Committee on Cancer (AJCC) staging system [13] was proposed in 2010 and subsequently endorsed by both the International Union for Cancer Control (UICC) [14] and the World Health Organisation (WHO) [15], which was primarily applied to classify pancreatic exocrine tumours. These two systems differ much in their respective definition of ‘T’ stage (Table 1), which results in corresponding differences when staging p-NETs. AJCC stage I disease encompasses all tumours that are confined to the pancreas, whereas ENETS stage I is restricted to tumours confined to the pancreas and <2cm in diameter; AJCC stage III disease refers to locally advanced tumours involving the superior mesenteric artery or coeliac axis, whereas ENETS stage III includes locally advanced tumours that may be resectable or unresectable [16]. Meanwhile, it is difficult to distinguish T2 and T3 AJCC stages as this requires the identification of peri-pancreatic soft tissue invasion, which is sometimes challenging [17, 18].

**Table 1:** ENETS and AJCC TNM staging systems for p-NETs

T/N/M	ENETS	AJCC
T1	Tumour limited to the pancreas, <2 cm in greatest diameter.	Tumour limited to the pancreas, ≤2 cm in greatest diameter.
T2	Tumour limited to the pancreas, 2-4 cm in greatest diameter.	Tumour limited to the pancreas, >2 cm in greatest diameter.
T3	Tumour limited to the pancreas, >4 cm in greatest diameter, or invading duodenum or common bile duct.	Tumour extends beyond the pancreas, but not involving the celiac axis or superior mesenteric artery.
T4	Tumour invades adjacent structures (stomach, spleen, colon or the wall of large vessels including coeliac axis or superior mesenteric artery).	Tumour involves the coeliac axis or superior mesenteric artery (unresectable tumour).
N0	No regional LN metastasis	No regional LN metastasis
N1	Regional LN metastasis	Regional LN metastasis
M0	No distant metastasis	No distant metastasis
M1	Distant metastasis	Distant metastasis
**Stage**		
Ia	NA	T1 N0 M0
Ib	NA	T2 N0 M0
IIa	NA	T3 N0 M0
IIb	NA	T1-3 N1 M0
III	NA	T4 N0-1 M0
IV	NA	Any T M1
Or		
I	T1 N0 M0	NA
IIA	T2 N0 M0	NA
IIB	T3 N0 M0	NA
IIIA	T4 N0 M0	NA
IIIB	Any T N1 M0	NA
IV	Any T Any N M1	NA

ENETS, European Neuroendocrine Tumour Society; AJCC, American Joint Committee on Cancer; TNM, tumour-node-metastasis; p-NETs, pancreatic neuroendocrine tumour; LN, lymph node; NA, not applicable.

Some studies have validated and compared the 2 TNM staging systems and their ability to prognosticate effectively in terms of the overall survival (OS) of p-NETs [16, 19-23], but few evaluated them for functional or non-functional tumours respectively. In the present study, undertaken at a single specialist centre in China, we investigated the prognostic value of the two TNM systems in patients with p-NETs, as a whole, and more interestingly in functional and non-functional groups separately, with a view to ascertaining any potential clinical benefits of using one system over the other in each of these two sub-groups.

## RESULTS

### Patient demographics and tumour characteristics

#### All p-NETs

As Table 2 showed, a total of 165 eligible patients were enrolled in our present study. A hundred and ten patients (66.7%) had functional p-NETs, while 55 cases (33.3%) were diagnosed as non-functional. Seventeen patients (10.3%) with asymptomatic non-functional p-NETs were detected incidentally by health examination. All patients underwent pancreatic surgery including 143 patients (86.7%) with radical resections and 22 (13.3%) with palliative or explorative operations. The clinical characteristics of all patients with p-NETs were clearly outlined in Table 3. Based on the available pathological information of all specimens, the WHO 2010 grading classification was only applied to 114 tumours - there were 62 NET G1, 35 NET G2, and 17 neuroendocrine carcinoma (NEC) G3. Patients had a median follow-up time of 49.7 months and a mean of 52.5 ± 32.5 months (range 5.9 to 135.9), with 41 (24.8%) dead ones.

**Table 2:** Functional sub-groups and surgical procedures undertaken for all p-NETs

Variables	n (%)
**Functional status(N=165)**	
Functional group	110 (66.7)
Insulinoma	99 (60.0)
Gastrinoma	5 (3.0)
VIPoma	2 (1.2)
ACTHoma	2 (1.2)
Glucagonoma	1 (0.6)
Pheochromocytoma	1 (0.6)
Non-functional group^*^	55 (33.3)
Nausea and vomiting	33 (60.0)
Abdominal pain and mass	28 (50.9)
Health examination	17 (30.3)
Jaundice	9 (14.5)
Gastrointestinal bleeding	7 (12.1)
Weight loss	4 (7.3)
**Surgical procedure(N=165)**	
Radical resection	143 (86.7)
LP	62 (37.6)
DP	42 (25.5)
PD	24 (14.5)
Others†	15 (9.1)
Palliative or explorative operation	22 (13.3)

*One patient might present two or more clinical manifestations; †Such as central pancreatectomy and total pancreatectomy; p-NETs, pancreatic neuroendocrine tumour; VIPoma, vasoactive intestinal polypeptidoma; ACTHoma, adrenocorticotropic hormone adenoma. LP, local resection of pancreatic tumour (enucleation included); DP, distal pancreatectomy; PD, pancreaticoduodenectomy.

**Table 3:** Characteristics of functional and non-functional sub-groups of p-NETs

Variables	All	Functional	Non-functional	P*
No. of patients	n=165	n=110	n=55	
Gender, n (%)				0.078
Male	75 (45.5)	44 (40)	31 (56.4)	
Female	90 (54.5)	66 (60)	24 (43.6)	
Age at diagnosis, yrs, mean±SD	46.6 ±14.2	43.3 ±14.5	52.4 ±11.6	0.021
Tumour size, cm, mean ± SD	3.0 ± 2.3	2.3 ± 2.4	5.1 ±1.9	0.015
Tumour location, n (%)				0.126
Head and/or uncinate	70 (42.4)	42 (38.2)	28 (50.9)	
Body and/or tail	95 (57.6)	68 (61.8)	27 (49.1)	
Lymph node invasion, n (%)	20 (12.1)	8 (7.3)	12 (21.8)	0.281
Distant metastasis, n (%)	16 (9.7)	6 (5.5)	10 (18.2)	0.136
WHO 2010 grade, n (%)†				0.425
NET G1	62 (54.4)	47 (41.2)	15 (13.2)	
NET G2	35 (30.7)	8 (7.0)	27 (23.7)	
NEC G3	17 (14.9)	7 (6.1)	10 (8.8)	
Main operations, n (%)‡				0.247
LP	62 (48.4)	50 (61.7)	12 (25.5)	
DP	42 (32.8)	22 (27.2)	20 (42.6)	
PD	24 (18.8)	9 (11.1)	15 (31.9)	
Dead at follow-up§	41 (24.8)	20 (18.2)	21 (38.2)	0.418
T stage by ENETS				0.125
T1	90	81	9	
T2	17	7	10	
T3	25	13	12	
T4	33	9	24	
Clinical stage by ENETS				0.351
I	78	69	9	
II	36	13	23	
III	35	22	13	
IV	16	6	10	
T stage by AJCC				0.495
T1	90	81	9	
T2	37	11	26	
T3	23	12	11	
T4	15	6	9	
Clinical stage by AJCC				0.276
I	106	86	20	
II	32	14	18	
III	11	4	7	
IV	16	6	10	

*P value indicates the comparison between functional and non-functional groups; †Total n=114, functional group n=62, non-functional group n=52; ‡Total n=128, functional group n=81, non-functional group n=47; §Total n=140, functional group n=90, non-functional group n=50; p-NETs, pancreatic neuroendocrine tumours; SD, standard deviation; WHO, World Health Organisation; NEC, neuroendocrine carcinoma; LP, local resection of pancreatic tumour (enucleation included); DP, distal pancreatectomy; PD, pancreaticoduodenectomy.

#### Functional vs. non-functional p-NETs

Also in Table 3, the mean age at diagnosis of patients with functional p-NETs was significantly lower than that of the non-functional group (*P*=0.021). Tumour diameter was notably smaller in the functional group than that of the non-functional group (*P*=0.015). Most functional p-NETs were of NET G1 grade, as compared to non-functional p-NETs which were mainly of NET G2 and NEC G3 grades according to the WHO grading system (47/62 *vs*. 37/52, *P*=0.425).

### Stages and OS by ENETS and AJCC

#### All p-NETs

According to the definitions of both systems (Table 1), there were 90 patients with T1 tumours, 17 T2, 25 T3 and 33 T4 by the ENETS 2006 system (Table 3), which corresponded to 78, 36, 35 and 16 cases with stages I to IV disease respectively. As for the AJCC 2010 criteria, there were 90 T1, 37 T2, 23 T3, and 15 T4 tumours, which resulted to 106, 32, 11 and 16 patients from stages I to IV respectively.

**Figure 1:**
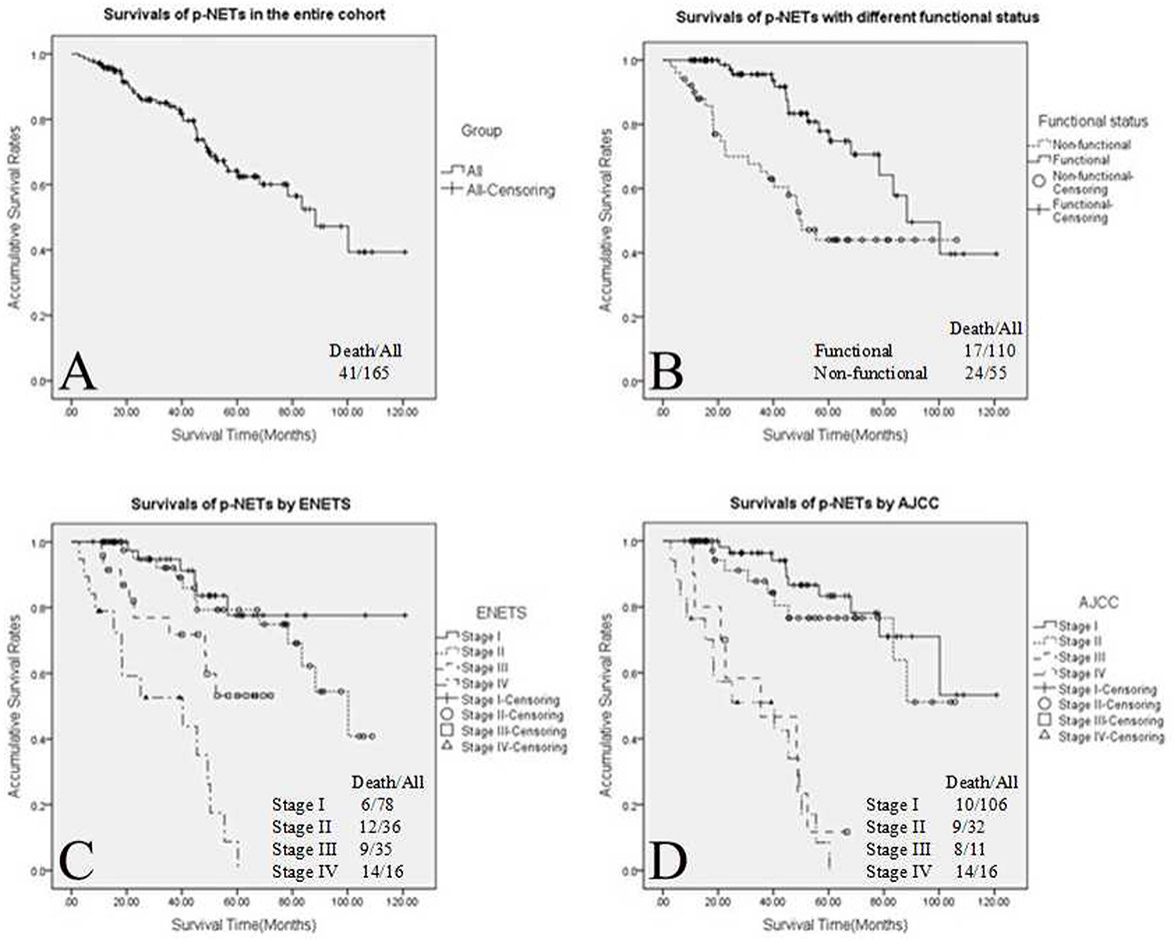
Kaplan–Meier curves of 5-year overall survival for all p-NETs : **(A)** all p-NETs, **(B)** functional sub-groups, **(C)** all p-NETs staged using the ENETS system and **(D)** all p-NETs staged using the AJCC system. P-NETs, pancreatic neuroendocrine tumours; ENETS, European Neuroendocrine Tumour Society; AJCC, American Joint Committee on Cancer.

The 5-year OS rate of the whole group was 62.4% (Figure 1A). Patients with functional p-NETs present a significantly higher OS than those with non-functional ones (*P*=0.001; Figure 1B). Using the ENETS staging system for the whole group of p-NETs (Figure 1C), OS differences were statistically significant when comparing stage I with stages III and IV (I *vs*. III, *P*=0.030; I *vs*. IV, *P*<0.001), as well as stage II with stages III and IV (II *vs*. III, *P*=0.048; II *vs*. IV, *P*<0.001); the differences between stage I and II were not significant (*P*=0.453). ENETS stage III patients had a significantly higher OS than those with stage IV p-NETs (*P*=0.003). As far as the AJCC staging system was applied for all p-NETs (Figure 1D), the OS differences between stage I and those in stages III and IV were statistically significant (I *vs*. III, *P*<0.001; I *vs*. IV, *P*<0.001); this was also the case comparing those in stage II and stages III and IV (II *vs*. III, *P*<0.001; II *vs*. IV, *P*<0.001). There was no difference in OS when comparing stage I with II, or stage III with IV (I *vs*. II, *P*=0.361; III *vs.* IV, *P*=0.601).

#### Functional vs. non-functional p-NETs

As Table 3 outlined, most functional p-NETs were of T1 stage (N_(ENETS) T1_=N_(AJCC) T1_=81). Patients with functional p-NETs were mainly grouped in AJCC stages I and II (N_stage I_=86, N_stage II_=14, respectively), or in ENETS stages I, II and III (N_stage I_=69, N_stage II_=13, N_stage III_=22, respectively). Most non-functional p-NETs were ENETS system T3 and T4 (N_T3_=12, N_T4_=24, respectively), and AJCC system T2 and T3 (N_T2_=26, N_T3_=11, respectively). Non-functional p-NETs were classified as ENETS system stages II and III (N_stage II_=23, N_stage III_=13, respectively), or as AJCC system stages I and II (N_stage I_=20, N_stage II_=18, respectively). Patients with Stage IV disease were equal in functional (N_(ENETS) stage IV_=N_(AJCC) stage IV_=6) and non-functional groups (N_(ENETS) stage IV_=N_(AJCC) stage IV_=10).

**Figure 2:**
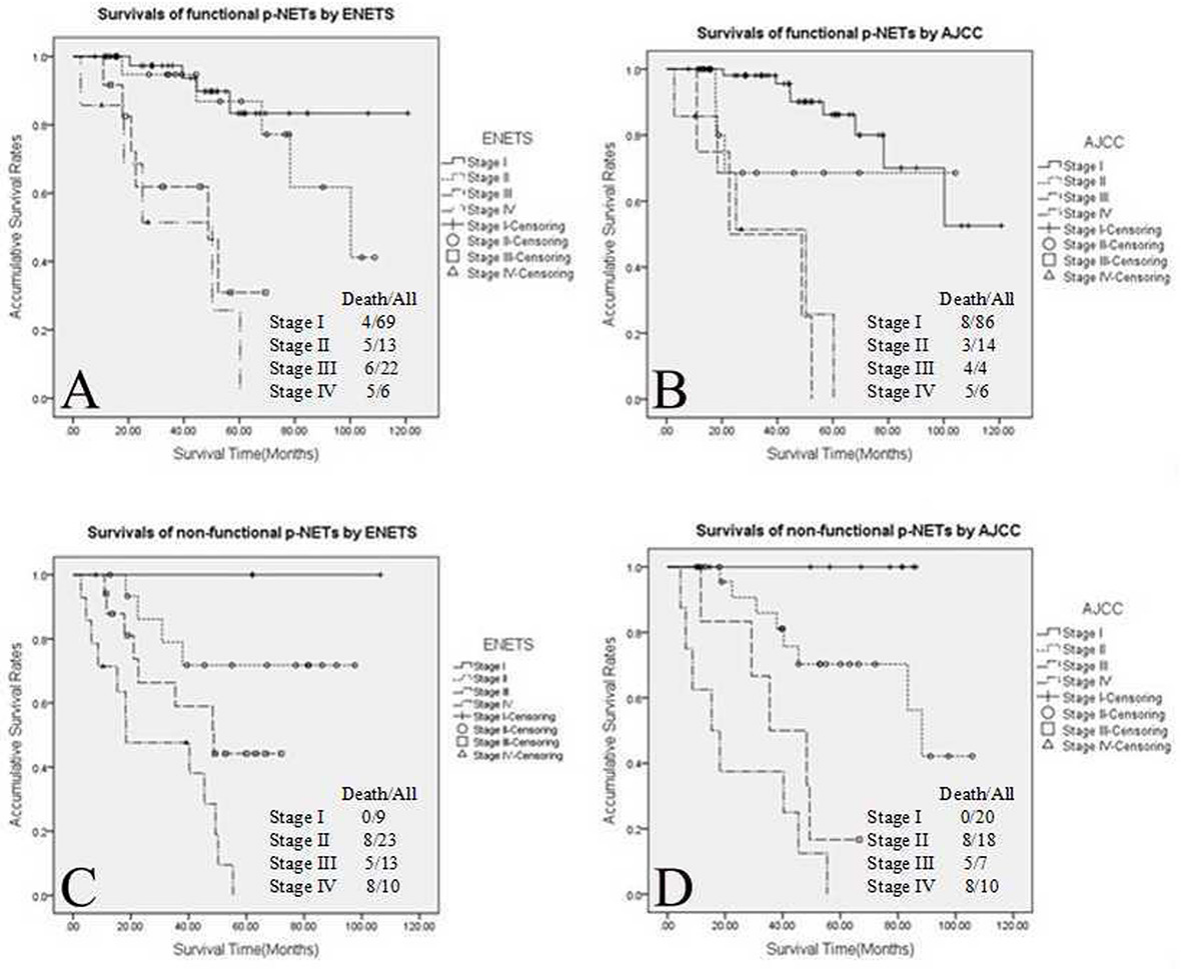
Kaplan–Meier curves of 5-year overall survival of the 2 functional sub-groups of p-NETs **(A)** Functional sub-group staged using the ENETS system, **(B)** functional sub-group staged using the AJCC system, **(C)** non-functional sub-group staged using the ENETS system and **(D)** non-functional sub-group staged using the AJCC system. P-NETs, pancreatic neuroendocrine tumours; ENETS, European Neuroendocrine Tumour Society; AJCC, American Joint Committee on Cancer.

When survival analyses of functional tumours were carried out using the ENETS classification (Figure 2A), the OS rate differences of patients with functional p-NETs were statistically significant when comparing stage I with stages III and IV (I *vs*. III, *P*<0.001; I *vs*. IV, *P*<0.001), as well as stage II and stages III and IV (II *vs*. III, *P*=0.009; II *vs*. IV, *P*<0.001); differences in OS of stage I with II or stage III with IV were not significantly different (I *vs*. II, *P*=0.409; III *vs*. IV, *P*=0.499). Using the AJCC criteria (Figure 2B), only patients with functional tumours in stage I showed a significantly better OS rate than those with stages III and IV tumours (I *vs*. III, *P*<0.001; I *vs*. IV, *P*<0.001), while there were no significant differences when comparing patients in stages I and II, stages II and III, stages II and IV and stages III and IV (all *P*>0.05). Similarly, using the ENETS system to compare patients with non-functional tumours (Figure 2C), stages I, II and III had a significantly better survival than those with stage IV tumours (I *vs*. IV, *P*=0.005; II *vs*. IV, *P*<0.001; III vs. IV, *P*=0.026). However, there were no OS differences when comparing patients in stage I with those in stages II and III, or between those in stages II and III (all *P*>0.05). Staging non-functional p-NET patients according to the AJCC system (Figure 2D), the differences in OS were statistically significant when comparing patients in stage I with those in stages III and IV (I *vs*. III, *P*=0.001; I *vs*. IV, *P*<0.001), as well as patients in stage II with those in stages III and IV (II *vs*. III, *P*=0.017; II *vs*. IV, *P*<0.001); whereas patients in stages I and II or stages III and IV did not show any significant differences in OS (*P*>0.05).

### Analyses of potential prognostic factors

#### All p-NETs

For the whole group of p-NETs (Table 4), patient age, tumour size, hormone status, surgical intent, WHO 2010 grade, stage by both ENETS and AJCC were all significantly associated with the OS of p-NETs on univariate analysis (all *P*<0.05), while patients gender, tumour location and main operation weren’t (all *P*>0.05). Further multivariate analyses indicated that only surgical intent, WHO 2010 grade, ENETS and AJCC stage were statistically valuable predictors of OS in p-NETs (Table 5).

**Table 4:** Univariate analysis of predictors of overall survival in patients with p-NETs

Variables	All (n=165)		Functional (n=110)		Non-functional (n=55)	
	HR (95%CI)	*P*	HR (95%CI)	*P*	HR (95%CI)	*P*
Gender						
Male	2.55	0.108	2.48	0.072	2.13	0.163
Female	(1.28-5.08)		(0.92-6.69)		(0.74-6.17)	
Age, years*						
<Median	0.50	0.048	0.64	0.375	0.53	0.215
≥Median	(0.25-1.00)		(0.24-1.72)		(0.19-1.45)	
Tumour size, cm*						
<Median	0.26	0.012	0.24	0.008	0.99	0.989
≥Median	(0.13-0.53)		(0.08-0.68)		(0.28-3.50)	
Tumour location						
Head and/or uncinate	0.52	0.065	0.34	0.039	0.82	0.681
Body and/or tail	(0.26-1.02)		(0.12-0.95)		(0.33-2.08)	
Hormone status†						
Functional	1.91	0.032	-	-	-	-
Non-functional	(1.36-2.68)					
ENETS clinical stage						
I and II	0.94	0.005	5.30	< 0.001	2.40	0.002
III and IV	(2.61-5.96)		(2.80-10.05)		(1.38-4.17)	
AJCC clinical stage						
I and II	0.07	0.014	0.05	< 0.001	0.13	0.001
III and IV	(0.03-0.16)		(0.02-0.17)		(0.04-0.40)	
WHO 2010 grade						
NET G1 and G2	0.11	< 0.001	0.07	< 0.001	0.16	0.014
NEC G3	(0.01-0.16)		(0.01-0.22)		(0.03-0.45)	
Surgical intent						
Radical	6.07	< 0.001	10.91	< 0.001	3.74	0.021
Palliative	(2.41-15.26)		(3.39-35.13)		(1.22-11.52)	
Main operation						
LP	1.20	0.084	0.63	0.045	0.83	0.105
DP and PD	(0.25-2.00)		(0.14-1.02)		(0.49-1.73)	

*The median value per each group was used for the respective analysis; †Hormone status was only analyzed for all p-NETs; p-NETs, pancreatic neuroendocrine tumours; HR, hazard ratio; CI, confidence interval; ENETS, European Neuroendocrine Tumour Society; AJCC, American Joint Committee on Cancer; WHO, World Health Organisation; NEC, neuroendocrine carcinoma; LP, local resection of pancreatic tumour (enucleation included); DP, distal pancreatectomy; PD, pancreaticoduodenectomy.

**Table 5:** Multivariate analysis of predictors of overall survival in patients with p-NETs

Variables	All (n=165)		Functional (n=110)		Non-functional (n=55)	
	HR (95%CI)	*P*	HR (95%CI)	*P*	HR (95%CI)	*P*
Age, years*						
< Median	1.32	0.413	-	-	-	-
≥ Median	(0.54-3.84)					
Tumour size, cm*						
< Median	2.18	0.59	3.14	0.935	-	-
≥ Median	(0.37-4.25)		(0.05-8.26)			
Tumour location						
Head and/or uncinate	-	-	1.12	0.724	-	-
Body and/or tail			(0.24-5.62)			
Hormone status†						
Functional	2.43	0.216	-	-	-	-
Non-functional	(0.35-5.48)					
ENETS clinical stage						
I and II	4.41	0.036	5.22	0.043	6.72	0.021
III and IV	(3.25-11.25)		(1.14-18.15)		(0.96-15.24)	
AJCC clinical stage						
I and II	5.24	0.047	7.65	0.029	9.62	0.016
III and IV	(1.21-15.25)		(0.06-21.16)		(1.86-11.25)	
WHO 2010 grade						
NET G1 and G2	5.24	0.012	6.21	0.026	5.14	0.036
NEC G3	(1.41-25.15)		(0.95-21.32)		(1.27-28.83)	
Surgical intent						
Radical	4.15	0.032	4.92	0.027	3.22	0.035
Palliative	(3.23-11.33)		(1.62-19.79)		(1.72-13.52)	
Main operation						
LP	-	-	2.20	0.802	-	-
DP and PD			(0.11-7.17)			

*The median value per each group was used for respective analysis; †Hormone status was only analyzed for all p-NETs; p-NETs: pancreatic neuroendocrine tumours; HR, hazard ratio; CI, confidence interval; ENETS, European Neuroendocrine Tumour Society; AJCC, American Joint Committee on Cancer; WHO, World Health Organisation; NEC, neuroendocrine carcinoma; LP, local resection of pancreatic tumour (enucleation included); DP, distal pancreatectomy; PD, pancreaticoduodenectomy.

#### Functional vs. non-functional p-NETs

Also in Table 4, the simultaneous univariate analyses for different function-status p-NETs revealed that tumour size and location, and the main operation performed were associated with the OS in the functional group (all *P*<0.05), but not in non-functional tumours (all *P*>0.05). We further calculated on multivariate analyses that, for both functional and non-functional p-NETs sub-groups, surgical intent, WHO 2010 grade, ENETS and AJCC stage were also significant prognostic factors (Table 5).

## DISCUSSION

TNM staging is a useful instrument for death-risk assessment and patient stratification that may facilitate effective management, if it accurately reflects the biological behavior and natural history of the cancer that it is being applied to [22]. However, currently, presence of these two TNM systems for p-NETs by ENETS and AJCC might have caused clinical confusion in disease stratification and patient management [17, 27]. Also, p-NETs are more indolent in their biological behaviors, with a corresponding better OS than pancreatic exocrine tumours [28, 29]. Therefore, evidence is essential for the development and application of a uniform TNM staging system for this group of tumours [30]. A comparative study in 2012 by Rindi et al. [22] concluded that although both TNM systems were independent predictors of OS for p-NETs, the ENETS staging system was superior to the AJCC manual, which demonstrated a more accurate predictive ability for each stage. This result has also been reported by some other subsequent studies [19, 20, 23]. In the present study, as we described before, when investigating all p-NETs, the analyses similarly indicated that the ENETS system could also more effectively discriminate the OS difference between stages III and IV (*P*=0.003) as compared to the AJCC system (*P*=0.601).

Although some previous studies have compared these 2 TNM staging systems for the OS of p-NETs, most of them just combined functional and non-functional tumours as a whole group for analyses when comparing the prognostic value of various staging systems [16, 20-22]. However, the heterogeneity of p-NETs may not permit the effective application of these systems to both functional and non-functional p-NETs [31-36]. Our present study for the first time evaluated both TNM systems for different function-status p-NETs separately. For functional sub-group of patients, the ENETS system could effectively discriminate OS differences between stage I and stages III and IV or between stage II and stages III and IV, but the AJCC system could only significantly discriminate OS between stage I and stages II and IV. With respect to the non-functional sub-group, the ENETS system could only effectively discriminate the OS differences between stage IV with stages I, II and III, while the AJCC system, on the other hand, could effectively discriminate OS differences between stage I and stages III and IV, as well as those between stage II stages III and IV. These phenomena provided us proofs that the ENETS system might be more practical for patients with functional tumours, while the AJCC system might be more useful when applied to non-functional p-NETs.

Several studies have confirmed the prognostic value of both TNM systems [16, 19-23]. In our study, the Cox Regression proportional hazards model analyses revealed that stage using both ENETS and AJCC systems, surgical intent and WHO 2010 grade were influential predictors of p-NETs with different functional status, which was similarly in accordance with our previous work [19, 37, 38]. Tumour size and hormone status were not found to be independent predictors of OS for p-NETs, unlike the reports by Halfdanarson et al. [3] and Bilimoria et al. [39]. Tumour size and location, and main operation were demonstrated to be statistically associated with OS of functional tumours on univariate analysis, but not for non-functional p-NETs [40]. Meanwhile, multivariate modeling demonstrated that although both TNM systems were independent predictors of OS, the AJCC 2010 staging manual showed a larger 95% CI for all patients and in the functional sub-group, while the ENETS 2006 staging system showed a larger 95% CI in non-functional tumours, indicative of a relatively imprecise predictive ability. Our demonstrations indicated that it might be more proper to respectively apply different TNM staging system to different functional-status p-NETs.

The highlight of our study was that it was the first attempt, using a large consecutive series of data from a single institution, at separately validating the 2 TNM staging systems in the functional and non-functional sub-groups of p-NETs to determine which one was more suitable in terms of its prognostic performance. While the potential significance of this study was its contribution to investigating the clinical application of the ENETS and AJCC systems in staging functional and non-functional p-NETs and the further development of these staging systems in p-NETs, it had some limitations. The study was retrospective and conducted at a single centre. Further multi-centre, prospective studies were warranted.

In summary, both TNM staging systems (ENETS and AJCC) were significant predictors of OS for patients with p-NETs, irrespective of functional status. The ENETS system have potential advantages when applied to all p-NETs and to the functional sub-group, while the AJCC manual might be more clinically applicable for non-functional p-NETs.

## MATERIALS AND METHODS

### Patient selection

This research was approved by the local ethics committee of West China Hospital, Sichuan University, in accordance with the general principles of the Helsinki Declaration [24]. Written informed consent was obtained on admission from all patients for their information to be used for research. Electronic or paper-based medical records of consecutive patients, who were all surgically treated and pathologically diagnosed as p-NETs between January 2002 and February 2014, were retrospectively reviewed. Patients, in whom there was only clinical suspicion but no postoperative pathological confirmation of p-NETs, were excluded from this study. Only four patients underwent regular postoperative medical treatment, who were also excluded in our research. Furthermore, five patients with hereditary syndromes, including multiple endocrine neoplasia type I, von Hippel-Lindau syndrome weren’t enrolled as well.

### Data collection

For included patients, demographics, medical history, clinical presentation, preoperative imaging, tumour location and size, histopathologic results (lymph node involvement, vascular invasion, presence of metastasis, immunohistochemical staining, mitotic count or Ki-67 positive index, etc.), surgical procedures undertaken, perioperative outcomes, in-hospital stay, postoperative pathology findings and follow-up data, were all systematically collected using a pre-defined proforma.

### Tumour characteristics

Tumours were clinically classified as functional p-NETs when patients presented typical and specific clinical manifestations related to hormone over-production: insulinoma (typical Whipple triad), gastrinoma (refractory peptic ulcer, diarrhoea, esophagitis), VIPoma (intractable diarrhoea, hypokalaemia), ACTHoma (Cushing syndrome), glucagonoma (migratory erythema, hyperglycaemia), etc. For patients who didn’t present typical clinical features related to hormone over-production were considered having non-functional p-NETs, regardless of the immunohistochemical staining of tumour specimens and laboratory evidence of hormone rise [25]. All groups were classified and analysed according to the 2 TNM staging systems by ENETS and AJCC. The 2010 WHO pathological grading system [26] was also applied wherever possible.

### Follow-up and survival

Follow-up was conducted mainly by telephone or outpatient clinic appointments. OS was calculated as the number of months from the date of operation to the date of last contact or time of death.

### Statistical analysis

All statistical analyses were performed using IBM SPSS 19.0 statistical software. Quantitative variables were reported as mean with standard deviation (SD) and compared using the Student’s t or the analysis of variance tests. Categorical variables were presented as numbers with their frequencies as proportions (%) and analysed using Chi-square test (or Fisher’s exact test). OS estimates and curves of relevant cases were generated and plotted using the Kaplan-Meier method and then compared using the log-rank test. Univariate and multivariate analyses were applied to explore possible prognostic factors in both functional and non-functional p-NETs, using the Cox Regression proportional hazards model. Hazard ratio (HR) with 95% confidence interval (CI) was calculated for each variable. A *P* value of less than 0.05 was considered to be statistically significant.

## Abbreviations

P-NETs: pancreatic neuroendocrine tumours
VIP: vasoactive intestinal peptide
ACTH: adrenocorticotropic hormone
TNM: tumour-node-metastasis
ENETS: European Neuroendocrine Tumour Society
AJCC: American Joint Committee on Cancer
UICC: International Union for Cancer Control
WHO: World Health Organisation
NEC: neuroendocrine carcinoma
OS: overall survival
SD: standard deviation
HR: hazard ratio
CI: confidence interval

## References

[1] Pape UF, Bohmig M, Berndt U, Tiling N, Wiedenmann B, Plockinger U (2004). Survival and clinical outcome of patients with neuroendocrine tumors of the gastroenteropancreatic tract in a german referral center. Ann N Y Acad Sci.

[2] Lepage C, Bouvier AM, Phelip JM, Hatem C, Vernet C, Faivre J (2004). Incidence and management of malignant digestive endocrine tumours in a well defined French population. Gut.

[3] Halfdanarson TR, Rabe KG, Rubin J, Petersen GM (2008). Pancreatic neuroendocrine tumors (PNETs): incidence, prognosis and recent trend toward improved survival. Ann Oncol.

[4] Ito T, Sasano H, Tanaka M, Osamura RY, Sasaki I, Kimura W, Takano K, Obara T, Ishibashi M, Nakao K, Doi R, Shimatsu A, Nishida T (2010). Epidemiological study of gastroenteropancreatic neuroendocrine tumors in Japan. J Gastroenterol.

[5] Yao JC, Hassan M, Phan A, Dagohoy C, Leary C, Mares JE, Abdalla EK, Fleming JB, Vauthey JN, Rashid A, Evans DB (2008). One hundred years after “carcinoid“: epidemiology of and prognostic factors for neuroendocrine tumors in 35,825 cases in the United States. J Clin Oncol.

[6] Modlin IM, Moss SF, Chung DC, Jensen RT, Snyderwine E (2008). Priorities for improving the management of gastroenteropancreatic neuroendocrine tumors. J Natl Cancer Inst.

[7] Turaga KK, Kvols LK (2011). Recent progress in the understanding, diagnosis, and treatment of gastroenteropancreatic neuroendocrine tumors. CA Cancer J Clin.

[8] Vagefi PA, Razo O, Deshpande V, McGrath DJ, Lauwers GY, Thayer SP, Warshaw AL, Fernández-Del Castillo C (2007). Evolving patterns in the detection and outcomes of pancreatic neuroendocrine neoplasms: the Massachusetts General Hospital experience from 1977 to 2005. Arch Surg.

[9] Zerbi A, Falconi M, Rindi G, Delle Fave G, Tomassetti P, Pasquali C, Capitanio V, Boninsegna L, Di Carlo V (2010). Clinicopathological features of pancreatic endocrine tumors: a prospective multicenter study in Italy of 297 sporadic cases. Am J Gastroenterol.

[10] Oberg K, Eriksson B (2005). Endocrine tumours of the pancreas. Best Pract Res Clin Gastroenterol.

[11] Triponez F, Dosseh D, Goudet P, Cougard P, Bauters C, Murat A, Cadiot G, Niccoli-Sire P, Chayvialle JA, Calender A, Proye CA (2006). Epidemiology data on 108 MEN 1 patients from the GTE with isolated nonfunctioning tumors of the pancreas. Ann Surg.

[12] Rindi G, Klöppel G, Couvelard A, Komminoth P, Körner M, Lopes JM, McNicol AM, Nilsson O, Perren A, Scarpa A, Scoazec JY, Wiedenmann B (2006). TNM staging of foregut (neuro)endocrine tumors: a consensus proposal including a grading system. Virchows Arch.

[13] Edge S, Byrd DR, Compton CC (2010). AJCC Cancer Staging Manual.

[14] Sobin L, Gospodarowicz MK, Wittekind C (2009). TNM Classification of Malignant Tumours.

[15] Bosman F, Carneiro F, Hruban RH, Theise ND (2010). WHO Classification of Tumours of the Digestive System.

[16] Strosberg JR, Cheema A, Weber J, Han G, Coppola D, Kvols LK (2011). Prognostic validity of a novel American Joint Committee on Cancer Staging Classification for pancreatic neuroendocrine tumors. J Clin Oncol.

[17] Kloppel G, Rindi G, Perren A, Komminoth P, Klimstra DS (2010). The ENETS and AJCC/UICC TNM classifications of the neuroendocrine tumors of the gastrointestinal tract and the pancreas: a statement. Virchows Archiv.

[18] Liszka L, Pajak J, Mrowiec S, Zielinska-Pajak E, Golka D, Lampe P (2011). Discrepancies between two alternative staging systems (European Neuroendocrine Tumor Society 2006 and American Joint Committee on Cancer/Union for International Cancer Control 2010) of neuroendocrine neoplasms of the pancreas. A study of 50 cases. Pathol Res Pract.

[19] Yang M, Zeng L, Zhang Y, Wang WG, Wang L, Ke NW, Liu XB, Tian BL (2015). TNM staging of pancreatic neuroendocrine tumors: an observational analysis and comparison by both AJCC and ENETS systems from 1 single institution. Medicine (Baltimore).

[20] Qadan M, Ma Y, Visser BC, Kunz PL, Fisher GA, Norton JA, Poultsides GA (2014). Reassessment of the current American Joint Committee on Cancer Staging System for pancreatic neuroendocrine tumors. J Am Coll Surgeons.

[21] Strosberg JR, Cheema A, Weber JM, Ghayouri M, Han G, Hodul PJ, Kvols LK (2012). Relapse-free survival in patients with nonmetastatic, surgically resected pancreatic neuroendocrine tumors: an analysis of the AJCC and ENETS staging classifications. Ann Surg.

[22] Rindi G, Falconi M, Klersy C, Albarello L, Boninsegna L, Buchler MW, Capella C, Caplin M, Couvelard A, Doglioni C, Delle Fave G, Fischer L, Fusai G (2012). TNM staging of neoplasms of the endocrine pancreas: results from a large international cohort study. J Natl Cancer Inst.

[23] Ellison TA, Wolfgang CL, Shi C, Cameron JL, Murakami P, Mun LJ, Singhi AD, Cornish TC, Olino K, Meriden Z, Choti M, Diaz LA, Pawlik TM (2014). A Single institution's 26-year experience with nonfunctional pancreatic neuroendocrine tumors: a validation of current staging systems and a new prognostic nomogram. Ann Surg.

[24] Crawley FP (2012). The Limits of the Declaration of Helsinki. In: Address to Scientific Session.

[25] Metz DC, Jensen RT (2008). Gastrointestinal neuroendocrine tumors: pancreatic endocrine tumors. Gastroenterology.

[26] Rindi G, Arnold R, Bosman FT, Capella C, Klimstra DS, Kloppel G, Bosman T, Canerio F, Hruban R, Theise ND (2010). Nomenclature and classification of neuroendocrine neoplasms of the digestive system In: WHO classification of tumours of the digestive system.

[27] Fesinmeyer MD, Austin MA, Li CI, De Roos AJ, Bowen DJ (2005). Differences in survival by histologic type of pancreatic cancer. Cancer Epidem Biomar.

[28] Jarufe NP, Coldham C, Orug T, Mayer AD, Mirza DF, Buckels JA, Bramhall SR (2005). Neuroendocrine tumours of the pancreas: Predictors of survival after surgical treatment. Digest Surg.

[29] Quirke P, Cuvelier C, Ensari A, Glimelius B, Laurberg S, Ortiz H, Piard F, Punt CJ, Glenthoj A, Pennickx F, Seymour M, Valentini V, Williams G (2010). Evidence-based medicine: the time has come to set standards for staging. J Pathol.

[30] Rossi G, Nannini N, Mengoli MC, Cavazza A (2010). Neuroendocrine tumors: what staging system?. Am J Surg Pathol.

[31] Shiba S, Morizane C, Hiraoka N, Sasaki M, Koga F, Sakamoto Y, Kondo S, Ueno H, Ikeda M, Yamada T, Shimada K, Kosuge T, Okusaka T (2016). Pancreatic neuroendocrine tumors: a single-center 20-year experience with 100 patients. Pancreatology.

[32] Kent RB, van Heerden JA, Weiland LH (1981). Nonfunctioning islet cell tumors. Ann Surg.

[33] Rindi G, Bordi C (2005). Endocrine tumours of the gastrointestinal tract: aetiology, molecular pathogenesis and genetics. Best Pract Res Clin Gastroenterol.

[34] Panzuto F, Nasoni S, Falconi M, Corleto VD, Capurso G, Cassetta S, Di Fonzo M, Tornatore V, Milione M, Angeletti S, Cattaruzza MS, Ziparo V, Bordi C (2005). Prognostic factors and survival in endocrine tumor patients: comparison between gastrointestinal and pancreatic localization. Endocr Relat Cancer.

[35] Saif MW (2011). Pancreatic neoplasm in 2011: an update. JOP.

[36] Bettini R, Boninsegna L, Mantovani W, Capelli P, Bassi C, Pederzoli P, Delle Fave GF, Panzuto F, Scarpa A, Falconi M (2008). Prognostic factors at diagnosis and value of WHO classification in a mono-institutional series of 180 non-functioning pancreatic endocrine tumours. Ann Oncol.

[37] Yang M, Ke NW, Zeng L, Zhang Y, Tan CL, Zhang H, Mai G, Tian BL, Liu XB (2015). Survival analyses for patients with surgically resected pancreatic neuroendocrine tumors by World Health Organization 2010 Grading Classifications and American Joint Committee on Cancer 2010 Staging Systems. Medicine (Baltimore).

[38] Yang M, Tian BL, Zhang Y, Su AP, Yue PJ, Xu S, Wang L (2014). Evaluation of the World Health Organization 2010 Grading System in surgical outcome and prognosis of pancreatic neuroendocrine tumors. Pancreas.

[39] Bilimoria KY, Talamonti MS, Tomlinson JS, Stewart AK, Winchester DP, Ko CY, Bentrem DJ (2008). Prognostic score predicting survival after resection of pancreatic neuroendocrine tumors - analysis of 3851 patients. Ann Surg.

[40] Yang M, Zeng L, Zhang Y, Su AP, Yue PJ, Tian BL (2014). Surgical treatment and clinical outcome of nonfunctional pancreatic neuroendocrine tumors: a 14-year experience from one single center. Medicine (Baltimore).

